# The Critical Role of the Bile Acid Receptor TGR5 in Energy Homeostasis: Insights into Physiology and Therapeutic Potential

**DOI:** 10.3390/ijms26146547

**Published:** 2025-07-08

**Authors:** Lucas Zangerolamo, Marina Carvalho, Helena C. L. Barbosa

**Affiliations:** Obesity and Comorbidities Research Center, University of Campinas—UNICAMP, Campinas 13083-970, Sao Paulo, Brazil

**Keywords:** bile acids, TGR5, GPCR, energy homeostasis, obesity

## Abstract

Over the past decades, bile acids have been recognized as important signaling molecules with significant roles in metabolic health and disease. Many of their beneficial effects are mediated through the activation of the Takeda G protein-coupled receptor 5 (TGR5), a G protein-coupled receptor ubiquitously expressed in both humans and animals. Upon activation, TGR5 stimulates adenylate cyclase, leading to increased cyclic adenosine monophosphate (cAMP) levels and subsequent activation of protein kinase A (PKA). PKA then phosphorylates and activates several downstream signaling pathways, including exchange protein directly activated by cAMP (EPAC), extracellular signal-regulated kinase 1/2 (ERK1/2), and protein kinase B (AKT). Through these pathways, TGR5 acts as a key molecular link between bile acid signaling and the regulation of energy metabolism. TGR5 activation has been associated with body weight loss in obese models, primarily by reducing food intake, enhancing thermogenesis in adipose tissue and muscle to increase energy expenditure, and improving insulin secretion. This review highlights recent advances in our understanding of TGR5 biology and critically examines its therapeutic potential, limitations, and controversies in the context of energy metabolism, offering new perspectives and opportunities for treating metabolic disorders.

## 1. Introduction

Bile acid (BA) synthesis is the primary pathway for cholesterol catabolism and is essential for maintaining whole-body cholesterol homeostasis after feeding. Functioning as natural detergents, BAs facilitate the absorption, transport, metabolism, and excretion of nutrients, drugs, and xenobiotics [1]. In recent decades, emerging research has identified BAs as important nutrient sensors and metabolic integrators, crucial for maintaining metabolic homeostasis [2]. Following feeding, circulating BA levels can increase micromolar concentrations through enterohepatic recirculation, spilling into systemic circulation [3]. BA signaling is primarily driven by the nuclear farnesoid X receptor (FXR) and the membrane-bound Takeda G protein-coupled receptor 5 (TGR5) [4]. While FXR plays a central role in maintaining BA homeostasis [5], it also exerts metabolic effects indirectly by inducing the expression of fibroblast growth factor 15/19 (FGF15/19), which acts as an endocrine signal regulating glucose and energy metabolism [6,7]. In contrast, TGR5 activation is more directly associated with metabolic regulation, including glucose handling, lipid utilization, and energy expenditure (EE) [8,9,10,11].

TGR5 was first identified by Takaharu Maruyama and colleagues in 2002 [12]. It is a rhodopsin-like, G protein-coupled receptor (GPCR) activated by conjugated and unconjugated BAs [13]. TGR5 is ubiquitously expressed in murine models and humans, and its activation has been highlighted by several studies as an important metabolic regulator, influencing body weight, EE, glucose metabolism, satiety, insulin resistance, liver steatosis, and atherosclerosis [8]. Beyond its metabolic roles, TGR5 contributes to non-metabolic functions, including protection against hepatic pathology, anti-inflammatory signaling, and the maintenance of proper biliary function [14]. Interestingly, emerging evidence suggests that TGR5 function may differ between sexes, as BA composition varies between male and female, potentially influencing its downstream effects [15,16,17].

In this review, we explore the properties of TGR5, its endogenous and exogenous agonists, and the signaling pathways mediated by TGR5 activation. We also examine its mechanisms of action as a potential therapeutic target for regulating energy metabolism, providing an update on recent advances in this field. Furthermore, we discuss the clinical trials involving TGR5 agonists, the challenges faced in therapeutic interventions, and the controversies in the literature regarding its molecular functions.

## 2. TGR5: Structure, Expression, and Mechanism of Action

The bile acid receptor TGR5, also known as G protein-coupled bile acid receptor 1 (GPBAR1) or membrane-type receptor for bile acids (M-BAR), is a GPCR expressed at low to moderate levels across most tissues and cell types [9,18]. The top eight organs with the highest baseline expression of TGR5 in humans, quantified in normalized Transcripts Per Million (nTPM) from RNA sequencing data, include adipose tissue, smooth muscle, gallbladder, breast, spleen, stomach, kidney, and heart [11].

The TGR5 gene is composed of a single exon, with a coding sequence of 993 base pairs that generate a cell-surface protein of 330 amino acids [13]. This protein consists of an extracellular N-terminus, an intracellular C-terminus, and seven transmembrane helices connected by intracellular and extracellular loops [9,12]. In humans, the TGR5 gene is located on chromosome 2q35, and its cDNA sequence shares over 80% homology with those of bovids, rabbits, and rodents, demonstrating high structural conservation among mammals [13].

The expression of the TGR5 (GPBAR1) gene is found to be regulated by several factors, including BA levels [19], epigenetic modifications [20], and environmental stimuli such as exercise [21]. However, the precise mechanisms governing TGR5 regulation remain poorly understood, warranting further investigation into its complex and dynamic nature.

Upon the binding of BAs to TGR5, a signal-amplifying mechanism characteristic of GPCRs is activated. This results in the release of a complex from TGR5 consisting of G-protein-αs, β, and γ subunits [10,13]. GDP is then exchanged for GTP on the G protein, leading to the dissociation into G-protein-αs and βγ dimers. The G-protein-αs subunit subsequently stimulates adenylyl cyclase, leading to an increase in cyclic adenosine monophosphate (cAMP) levels and activation of protein kinase A (PKA), thereby initiating further downstream signaling pathways [10,13]. Additionally, TGR5 stimulation has been reported to activate the exchange protein directly activated by cAMP (EPAC) [22,23]. EPAC activation via TGR5 is also known to activate the protein kinase B (AKT) signaling pathway. Once activated, EPAC interacts with the small GTPase Rap-1, promoting the activation of phosphoinositide 3-kinase (PI3K). PI3K catalyzes the production of phosphatidylinositol-3,4,5-trisphosphate (PIP3), which recruits AKT to the plasma membrane, where it undergoes phosphorylation and activation [11].

**Figure 1:**
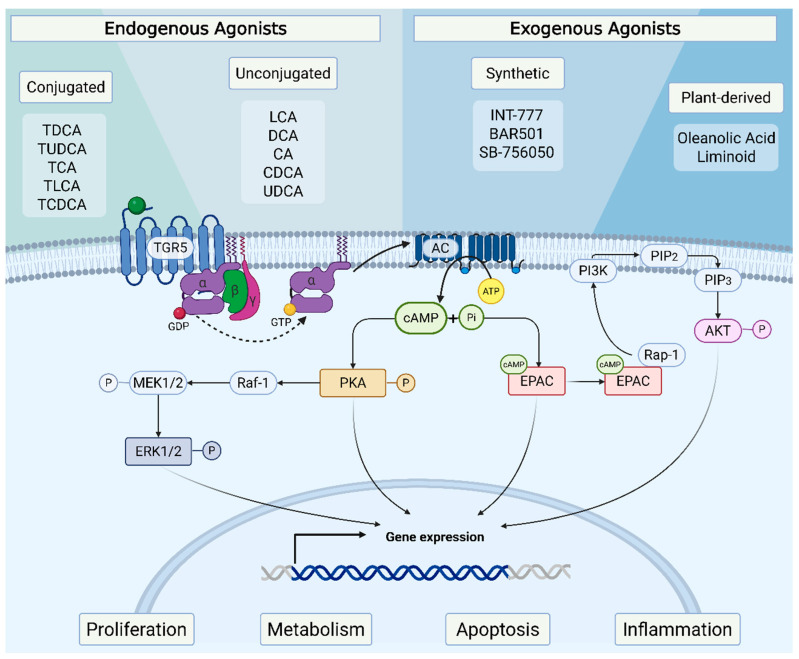
Schematic representation of canonical signaling pathways downstream of TGR5 activation and its agonists. TGR5 is a G protein-coupled receptor widely expressed in various tissues. It can be activated by both endogenous and exogenous agonists. Endogenous agonists include conjugated bile acids (e.g., TDCA, TUDCA, TCA, TLCA, and TCDCA) and unconjugated bile acids (e.g., LCA, DCA, CA, CDCA, and UDCA). Exogenous agonists include synthetic compounds such as INT-777, BAR501, and SB-756050, as well as plant-derived molecules like oleanolic acid and liminoids. Upon the binding of bile acids or synthetic agonists, TGR5 undergoes a conformational change that activates the associated G protein. This activation triggers the exchange of GDP for GTP on the G-protein-αs subunit, which then dissociates from the βγ subunits and stimulates adenylate cyclase. Activated adenylate cyclase catalyzes the conversion of ATP to cAMP, leading to an intracellular accumulation of this second messenger. The rise in cAMP concentration activates two main downstream effectors: PKA and EPAC. Activated PKA phosphorylates various target proteins, including ERK1/2, thereby modulating pathways involved in cell growth, differentiation, and metabolism. EPAC is directly activated by cAMP and functions as a guanine nucleotide exchange factor for small GTPases such as Rap-1. Activation of Rap-1 by EPAC indirectly promotes the phosphorylation of AKT, a key regulator of cell survival, proliferation, and glucose metabolism. TGR5: Takeda G protein-coupled receptor 5, TDCA: Taurodeoxycholic acid, TUDCA: Tauroursodeoxycholic acid, TCA: Taurocholic acid, TLCA: Taurolithocholic acid, TCDCA: Taurochenodeoxycholic acid, LCA: Lithocholic acid, DCA: Deoxycholic acid, CA: Cholic acid, CDCA: Chenodeoxycholic acid, UDCA: Ursodeoxycholic acid, INT-777: Synthetic TGR5 agonist, BAR501: Synthetic TGR5 agonist, SB-756050: Synthetic TGR5 agonist, cAMP: Cyclic adenosine monophosphate, PKA: Protein kinase A, EPAC: Exchange protein directly activated by cAMP, ERK1/2: Extracellular signal-regulated kinase 1/2, AKT: Protein kinase B. Created in Biorender. Helena Barbosa. (2025) https://BioRender.com (accessed on 8 May 2025).

Additionally, BAs binding to TGR5 can activate the mitogen-activated protein kinase (MAPK)/extracellular signal-regulated kinase (ERK) cascade [8,24]. This occurs through a cAMP-dependent mechanism, where PKA phosphorylates Raf-1, leading to the sequential activation of MAPK/ERK1/2 (MEK1/2) and ERK1/2 [25]. Overall, downstream signaling mediated by TGR5 is involved in gene transcription modulation, influencing cell proliferation, survival, apoptosis, inflammation, and metabolism. The key signaling pathways activated by TGR5 are summarized in Figure 1.

Comparing TGR5’s membrane-initiated signaling via cAMP and kinase cascades with FXR-mediated responses reveals clear differences in their underlying mechanisms: FXR is a nuclear receptor that modulates gene transcription upon ligand binding [26], whereas TGR5 triggers rapid, non-genomic effects through second messenger pathways. These distinct modes of action underscore TGR5’s complementary and potentially unique therapeutic relevance in metabolic diseases.

## 3. TGR5 Agonists

TGR5 agonists can be broadly categorized into two main groups: endogenous and exogenous compounds [27]. Endogenous agonists primarily consist of BAs, which can be further classified into unconjugated and conjugated forms. In contrast, exogenous agonists include semisynthetic BAs and plant-derived compounds [28].

Among unconjugated BAs, the potency of TGR5 activation follows this order: lithocholic acid (LCA) > deoxycholic acid (DCA) > chenodeoxycholic acid (CDCA) > cholic acid (CA) > ursodeoxycholic acid (UDCA) [9,27,29]. Additionally, conjugation of BAs with taurine enhances their TGR5 agonistic potency [29,30], making taurolithocholic acid (TLCA), taurochenodeoxycholic acid (TCDCA), taurodeoxycholic acid (TDCA), tauroursodeoxycholic acid (TUDCA), and taurocholic acid (TCA) additional TGR5 activators.

Considering both conjugated and unconjugated BAs, LCA and TLCA are the most potent endogenous TGR5 agonists [14], with EC50 values of 0.53 μM for LCA and 0.33 μM for TLCA [31]. Furthermore, certain steroid hormone intermediates, such as pregnandiol and 5α-pregnandione, have been shown to modulate TGR5 activity [29]. Table 1 summarizes the EC50 values of the principal TGR5 agonists discussed in this review.

Beyond endogenous ligands, semisynthetic agonists have also been developed to selectively activate TGR5, including INT-777 (Intercept Pharmaceuticals), which possesses micromolar potency and induces 166% of the cAMP synthesis effect observed with LCA [31,33]; BAR501 (BAR Pharmaceuticals), a UDCA derivative [34]; and SB-756050 (GlaxoSmithKline) [35]. Regarding plant-derived activators, obacunone, a limonoid present in *Citrus*, can stimulate TGR5 activity in a dose-dependent manner [37]. Moreover, the active triterpenoid oleanolic acid, found in olive leaves, has also been described as capable of signaling via TGR5 activation [38]. A summary of TGR5 agonists and the signaling pathways activated by them is presented in Figure 1.

## 4. TGR5 in Energy Homeostasis Regulation

### 4.1. Hypothalamus

The hypothalamus has been highlighted as a key target of BAs signaling associated with metabolic regulation, as it is known to control EE thermogenesis, and food intake [39]. BAs, including TGR5 agonists such as LCA, CA, CDCA, UDCA, and TUDCA, are present in the hypothalamus [40]. Notably, a specific increase in taurine-conjugated species, including TUDCA, UDCA, TCDCA, and TDCA, has been observed after feeding [41]. It is known that unconjugated BAs can diffuse through the blood–brain barrier, while conjugated BAs may require active transport into the brain by specific BAs transporters, such as the organic anion transporting polypeptides, the organic anion transporter, and the apical sodium-dependent bile acid transporter [42,43]. Additionally, BAs can be directly synthesized in the brain from cholesterol through the action of sterol 24-hydroxylase (CYP46A1), an enzyme primarily expressed in neurons [44]. Notably, BA levels in the brain correlate with their circulating levels [45].

TGR5 is highly expressed in the hypothalamus [40,41,46,47,48], with particularly abundant expression in the mediobasal hypothalamus [40,49], a key region that integrates peripheral signals, such as hormones, nutrient availability, and metabolic status, with neural inputs [50]. This integration plays a crucial role in regulating metabolic functions, body weight, and appetite [39]. Although TGR5 expression in the hypothalamus remains unchanged in obesity, in diet-induced obese (DIO) mice, hypothalamic BA levels are reduced, while total plasma BA concentrations remain unchanged [40]. Specifically, significant decreases in DCA, TDCA, and TCA were detected in the hypothalamus of DIO mice [40], suggesting that TGR5 activation may be impaired in obesity.

Intracerebroventricular (icv) infusion of CDCA reduces body weight gain, food intake, and improves insulin sensitivity in DIO mice [40]. Similar effects were observed after central infusion of a mix of BAs acting as TGR5 agonists, containing TCA, GCA, DCA, and CA [40]. Moreover, DIO mice treated with CDCA in the brain displayed higher EE and increased adrenoreceptor gene expression in white adipose tissue (WAT) and brown adipose tissue (BAT) [40].

Castellanos-Jankiewicz and colleagues demonstrated that DIO mice with TGR5 knockout, specifically in the mediobasal hypothalamus, gained more weight and exhibited hyperphagia compared to controls. These mice also showed reduced expression of thermogenic markers in BAT, including uncoupling protein 1 (UCP1), features associated with changes in sympathetic nervous system (SNS)-dependent thermogenesis, which favors the obese phenotype [40].

In line with these findings, another study showed that following a meal, BAs reach the brain and trigger a TGR5-mediated negative feedback loop regulating satiety in response to feeding [41]. In this study, both peripheral and central administration of a BA mix containing TGR5 agonists, as well as the TGR5-specific activator INT-777, led to an anorexigenic effect in wild-type mice by downregulating the orexigenic neuropeptides agouti-related peptide (AgRP) and neuropeptide Y (NPY). Conversely, the deletion of TGR5, whether whole-body, neuron-specific, or restricted to AgRP/NPY neurons, abolished BA-induced satiety [41]. Additionally, our group identified that TUDCA administration reduces AgRP and NPY expression and food intake in the hypothalamus of streptozotocin-induced Alzheimer’s disease mice model [51], effects that were also observed in wild-type mice [52]. Furthermore, DCA administration also inhibits food consumption in a TGR5-dependent manner in rats [53], reinforcing the role of TGR5 in the regulation of satiety.

To gain further insight into the hypophagic effect of TGR5 activation, a recent study showed that dietary supplementation and intranasal administration of oleanolic acid, a plant analog of BA, suppress appetite in mice by activating the TGR5/cAMP signaling pathway in the hypothalamus by inhibiting the expression and secretion of AgRP [52]. In vitro treatment of the mouse hypothalamic cell line N38 with oleanolic acid activates TGR5 and reduces AgRP gene and protein expression [52].

While TGR5 activation in the hypothalamus plays a key role in food intake suppression by reducing the synthesis and release of orexigenic neuropeptides, satiety induced by BAs is also mediated via vagal afferent pathways through TGR5 activation [53]. This study demonstrates that BAs induce satiety by activating TGR5-expressing vagal afferent neurons, which transmit signals to the hypothalamus. Notably, BAs act synergistically with cholecystokinin (CCK) to enhance anorexigenic signals in the hypothalamus and promote satiety [53].

It is noteworthy that, in addition to BAs reducing appetite, some BAs have been shown to upregulate the gene and protein expression of TGR5 in the hypothalamus and hypothalamic cell lines, such as TUDCA and oleanolic acid [52]. This suggests that, beyond acting as TGR5 activators, BAs may also serve as inducers of TGR5 expression. However, the specific mechanism behind this remains unclear.

Altogether, these findings suggest that TGR5 activation suppresses food intake by reducing orexigenic mRNA levels and transiently inhibiting the release of orexigenic neuropeptides in AgRP/NPY neurons. Additionally, TGR5 acts synergistically with CCK to activate anorexigenic neurons in the hypothalamus, thereby enhancing satiety.

### 4.2. Brown Adipose Tissue (BAT)

BAT is a remarkable organ with the capacity to dissipate energy as heat through thermogenesis, thereby playing a pivotal role in maintaining energy homeostasis. BAT thermogenesis exerts a crucial role in chronic cold adaptation, and its activation protects against obesity and various other comorbidities associated with metabolic disorders [54]. The discovery of functional BAT in adult humans significantly increased interest in thermogenesis [55].

While cold exposure is the primary activator of thermogenesis, a process driven by adrenergic receptor signaling that activates PKA and enhances UCP1 activity to generate heat, BAs can also enhance thermogenic activity in BAT by activating the TGR5 receptor [56,57,58,59,60].

In BAT, TGR5 activation by BAs elevates intracellular levels of the second messenger cAMP. This rise in cAMP stimulates type 2 deiodinase (D2), an enzyme that converts the inactive thyroid hormone thyroxine (T4) into its active form, 3-5-3′-triiodothyronine (T3). This mechanism not only increases BAT activity and enhances EE but also promotes EE in human skeletal muscle [56].

The regulation of D2 expression is intrinsically linked to TGR5 activation, as D2 is primarily controlled through the cAMP–PKA pathway and a functional cAMP-responsive element (CRE) [61]. Notably, TGR5 is the only GPCR known to respond to BAs by producing cAMP and activating PKA signaling [62]. The coexpression of TGR5 and D2 mRNAs in mouse BAT further underscores the functional relationship between these molecules, highlighting the critical role of TGR5 in driving D2-mediated BAT activation [56].

These findings are further supported by Watanabe and colleagues, who demonstrated that a diet supplemented with CA reduced adiposity and enhanced EE in DIO mice [56]. Interestingly, these effects were specifically attributed to TGR5 activation, as the thermogenic response induced by CA was absent in the presence of an FXR agonist, another receptor with high affinity for CA [56].

Furthermore, CA administration in mice alters BA composition by increasing the total and serum BA content, specifically elevating TCA [57], a potent TGR5 agonist [56]. This increase may be key to the anti-metabolic syndrome effects of CA administration via TGR5 activation.

Expanding on these observations, other BAs, such as CDCA, have also been shown to promote metabolic benefits. Dietary CDCA supplementation prevents body weight gain and induces significant weight loss in DIO mice, effects associated with increased expression of thermogenic markers, including UCP1, PR domain containing 16 (PRDM16), and peroxisome proliferator-activated receptor gamma coactivator 1-alpha (PGC1α) [58]. Similarly, CA and CCA supplementation in high-fat diet (HFD)-fed mice led to the upregulation of genes involved in EE regulation, such as D2 and UCP1 [56]. Moreover, CA-fed mice exhibited a more accentuated HF-induced reduction in the respiratory quotient, along with improved insulin sensitivity [56].

In addition to CDCA, other BAs, such as TUDCA, have been reported to enhance BAT thermogenesis and EE through mechanisms potentially involving TGR5 activation. The bile acid TUDCA has also been shown to increase D2 activity and T3 production in MSTO-211H cells, which endogenously express D2 [63]. While in murine brown adipocytes, TUDCA enhanced D2 expression and activity, oxygen consumption, and thermogenic markers [63]. These effects are attributed to its chemical chaperone activity and may depend on TGR5 activation, as BAs regulate D2 expression and T3 production through TGR5 activation. Moreover, our group has demonstrated that TUDCA enhances EE in Alzheimer’s disease [51] and aged [64] mice models. Given the well-established role of TGR5 activation in enhancing EE and the evidence supporting TUDCA’s ability to do the same, further studies are warranted to confirm whether TUDCA’s effects are indeed mediated through TGR5 activation.

Beyond its effects in rodent models, BAs have also been shown to influence BAT activity in humans [59]. Broeders and colleagues demonstrated that a 2-day CDCA treatment increased BAT activity and whole-body EE in healthy female subjects under thermoneutral conditions. In vitro, CDCA and TGR5 agonists enhanced mitochondrial uncoupling and D2 expression in human brown adipocytes [59]. Notably, this effect was exclusive to brown adipocytes and absent in white adipocytes. Overall, these findings further support BAs as promising modulators of energy metabolism and highlight their potential role in stimulating BAT in humans via TGR5 activation.

### 4.3. White Adipose Tissue (WAT)

While the effects of TGR5 activation in BAT have been extensively studied, its role in WAT remains less explored. Nonetheless, emerging evidence suggests that TGR5 signaling may also influence WAT function, particularly in the context of metabolic adaptations.

Velazquez-Villegas and colleagues demonstrate that TGR5 is essential for cold-induced beiging of subcutaneous white adipose tissue (scWAT), enhancing β-oxidation and mitochondrial function [8]. Using an adipose tissue-specific TGR5^−/−^ model (TGR5^Adipoq−/−^), the authors showed that fat-specific deletion of TGR5 impairs beiging when mice are exposed to 8 °C [8]. Moreover, adipocytes derived from the stromal vascular fraction of scWAT in wild-type mice and treated with INT-777 displayed increased expression of beiging markers, along with enhanced respiratory capacity. A similar effect was observed in the 3T3-L1 mouse adipocyte cell line, where TGR5 activation induced the expression of several beiging markers. Increased levels of UCP1 and PGC1α were also reported in both mice and 3T3-L1 cells treated with the synthetic TGR5 agonist BAR501 [65]. Additionally, stimulation of 3T3-L1 cells with LCA triggered an increase in beiging markers, which was blunted when TGR5 was silenced [8].

Beiging of WAT is a process by which white adipocytes acquire thermogenic properties similar to those of brown adipocytes. This conversion is characterized by the upregulation of UCP1, increased mitochondrial content, and enhanced EE [66,67]. Beiging can be trigged by several stimuli, including β-adrenergic activation, cold exposure, and specific hormonal or pharmacological signals, contributing to improved metabolic health by promoting energy dissipation rather than storage [66].

Even under thermoneutral conditions, where the sympathetic drive to the fat is minimal, TGR5 activation by the selective bile acid mimetic INT-777 is enough to trigger the beige remodeling program in mouse scWAT, regardless of external factors that typically stimulate the SNS [8], suggesting that this mechanism is independent of the sympathetic activation.

In humans, treatment with INT-777 in a human pre-adipocyte cell lineage enhanced the beige remodeling phenotype by upregulating key beiging markers, including UCP1, PGC1α, PRDM16, and cell death-inducing DNA fragmentation factor alpha-like effector A (CIDEA). These findings suggest that TGR5 activation in white human adipocytes promotes beige cell remodeling [8].

While TGR5 activation in human white adipocytes is promising, certain aspects remain unclear and warrant further investigation. A study has shown that TGR5 expression in human scWAT is positively correlated with obesity but decreases with weight loss [68]. One possible explanation for this upregulation during obesity is a compensatory mechanism to enhance beiging, as obesity has been linked to impaired beiging [69]. Additionally, the positive correlation between resting metabolic rate and TGR5 expression in human scWAT [68] further supports the idea that increased TGR5 levels in obesity may serve to promote beiging and enhance EE.

Beyond its role in beiging, TGR5 activation in WAT has been shown to exert anti-inflammatory effects, which may further contribute to metabolic homeostasis. Obese mice lacking TGR5 specifically in macrophages exhibit exacerbated adipose tissue inflammation, primarily due to an increase in pro-inflammatory M1 macrophages [70]. In contrast, TGR5 activation by INT-777 reduces macrophage infiltration and improves obesity-induced insulin resistance, a process mediated by the AKT-dependent activation of mammalian target of rapamycin complex 1 (mTORC1) [70]. These findings highlight an additional mechanism by which TGR5 signaling supports metabolic health, further emphasizing its potential as a therapeutic target.

While significant progress has been made in understanding how TGR5 activation influences WAT function, several gaps remain, particularly regarding its functions and mechanisms in humans. The evidence indicating a compensatory upregulation of TGR5 in obesity presents important questions about its physiological relevance and therapeutic potential. Future studies are needed to clarify whether pharmacological activation of TGR5 in WAT could serve as a viable strategy for metabolic disease intervention.

### 4.4. Skeletal Muscle

Skeletal muscle constitutes nearly 40% of human body mass. It fulfills support and locomotion functions and is the largest glucose-metabolizing organ, making this tissue essential for maintaining glucose homeostasis and promoting energy balance [71]. Given its central role in whole-body metabolism, skeletal muscle may represent an important target for TGR5 activation, particularly in the context of metabolic diseases where enhancing glucose uptake and EE is beneficial.

One of the most studied effects of TGR5 in skeletal muscle is its role in regulating the myogenic process and promoting muscle hypertrophy [21,72]. Its expression in skeletal muscle can be modulated by contractile activity, the unfolded protein response (UPR), and exercise [21]. Several studies have shown that TGR5 activation by LCA enhances AKT-mTORC1 pathway signaling [73], which in turn accelerates protein synthesis, cell differentiation, skeletal muscle regeneration following injury, and hypertrophy [72]. Notably, TUDCA administration in aged mice has been shown to increase skeletal muscle mass [64]. These findings are particularly relevant for energy homeostasis, as lean mass is directly proportional to EE [74].

TGR5 stimulation in skeletal muscle can also effectively regulate EE. Unlike rodents, adult humans have very little BAT [75], making skeletal muscle crucial for energy balance, as it is the predominant site of thermogenesis and insulin-induced glucose disposal [76,77]. As in BAT, TGR5 can promote the activation of D2 in skeletal muscle, which converts T4 into T3 and enhances the expression of key metabolic genes, including UCP1 and PGC-1α [10,76,78,79]. Watanabe and colleagues demonstrated that TGR5 activation by BAs, such as CA, DCA, TCA, and CDCA, induces a dose-dependent increase in D2 activity and oxygen consumption in human skeletal muscle myoblasts [56].

TGR5 agonist in skeletal muscle has also been associated with ameliorated insulin sensitivity in muscle tissue, leading to enhanced insulin-stimulated glucose uptake in the skeletal muscles of DIO mice and improved glucose homeostasis [78]. In this study, the authors developed and characterized a potent, selective TGR5 agonist called 4-phenoxynicotinamide (MN6). MN6 was able to improve insulin responsiveness in DIO mice and in the murine C2C12 myotubes through the cAMP/PKA pathway (31295453). Moreover, muscle-specific TGR5 overexpression improved glucose utilization and clearance in HFD-fed mice, activated glycolytic flux, and increased the respiratory exchange ratio [80].

A plant-derived compound called obacunone, a limonoid primarily found in citrus, can also act on skeletal muscle through TGR5 [37]. In this study, the authors reported that dietary obacunone intake significantly suppressed hyperglycemia and increased gastrocnemius and quadriceps muscle mass in obese KKAy mice, likely due to the stimulation of TGR5 transcriptional activity by obacunone. This further supports the role of TGR5 in promoting muscle mass in different experimental models.

### 4.5. Intestine

Beyond its well-established role in digestion and nutrient absorption, the intestine also functions as a dynamic endocrine organ that contributes to systemic energy homeostasis [81]. This aspect becomes particularly relevant in the context of TGR5 signaling. Following the discussion of TGR5 activation in classical metabolic tissues, it is important to highlight the contribution of the gut to energy regulation. One of the key metabolic effects of intestinal TGR5 activation is the stimulation of enteroendocrine L-cells to secrete glucagon-like peptide-1 (GLP-1), a potent incretin hormone known to enhance insulin secretion, suppress appetite, and improve glucose tolerance [33,78,82,83,84,85,86,87].

The mechanism associated with TGR5-induced GLP-1 secretion by BA, such as LCA and DCA, involves the activation of adenylate cyclase and subsequent elevation of cAMP levels, which activate PKA and EPAC to promote exocytosis of GLP-1-containing granules [82,88]. TGR5 activation in the murine enteroendocrine cell line STC-1 promotes GLP-1 secretion in a dose-dependent manner, while its knockdown by RNA interference abrogates this effect [82]. Similar outcomes were demonstrated in the murine L-cell line GLUTag and primary murine colon cultures, where LCA, TLCA, and DCA triggered GLP-1 secretion, along with the human enteroendocrine cell line NCI-H716 [33]. Furthermore, transfection of STC-1 cells with a plasmid to specifically upregulate TGR5 significantly enhanced GLP-1 secretion [82]. These findings establish TGR5 as a crucial mediator of BA-induced GLP-1 release.

The ability of TGR5 activation by BA to induce GLP-1 secretion has been further expanded by different studies, which demonstrated that TGR5 is co-localized with GLP-1 on the basolateral side of intestinal L-cells [83]. Activation of TGR5 by the agonist INT-777 also promotes GLP-1 secretion in enteroendocrine L-cells of the intestinal epithelium [33], thereby improving liver and endocrine pancreas function and ameliorating glucose metabolism in obese mice. On the other hand, TGR5 deficiency leads to the complete loss of BA-stimulated GLP-1 release [83,84].

Interestingly, TGR5 activation by a BA mix (containing CA, DCA, and CDCA in both unconjugated and taurine- and glycine-conjugated forms) [83] or oleanolic acid [88] has been linked to peptide YY (PYY) secretion, a gut hormone released postprandially with potent appetite-reducing activity [89]. This suggests that TGR5 activation in the gut may not only influence gastrointestinal function but also impact food-related behavior. Consistent with this, TGR5 deficiency resulted in the loss of BA-stimulated PYY secretion [83]. Mechanistically, TGR5-mediated PYY secretion appears to be mediated by the cAMP/EPAC signaling pathway [88].

TGR5 activation in the gut has also been associated with the beneficial effects of gastric bypass in obese rats [90]. Gastric bypass surgery induced changes in taurine metabolism by modulating gut microbiota, which led to an increased content of taurine-conjugated BA species, including TCA, TDCA, TUDCA, and TCDCA. These changes resulted in improved glucose homeostasis, better lipid control, enhanced adaptive thermogenesis, and mitigation of fatty liver disease. In contrast, administering an antibiotic cocktail to deplete the gut microbiota in obese rats following gastric bypass surgery abolished their beneficial effects on energy balance and glucolipid metabolism. Furthermore, microbiota transplantation from obese rats subjected to gastric bypass to HFD-fed rats enhanced TGR5 expression in BAT, WAT, and the gut [90]. Another study also demonstrated that TGR5 is essential for mediating the anti-obesity and anti-hyperglycemic effects, while also attenuating hepatic steatosis in mice undergoing vertical sleeve gastrectomy [84].

TGR5 activation has also emerged as a promising therapeutic target in inflammatory bowel disease (IBD), particularly in the context of obesity-associated intestinal inflammation. Obesity exacerbates IBD pathology through increased pro-inflammatory cytokines and impaired gut barrier function. Activation of TGR5 helps mitigate these effects by promoting anti-inflammatory signaling pathways, reducing macrophage and immune cell activation [91], and enhancing epithelial barrier integrity [92]. These combined effects position TGR5 as a crucial link between metabolic status and intestinal immune regulation, offering potential for targeted interventions in obesity-related IBDs.

In summary, these studies collectively position intestinal TGR5 as a central integrator of systemic energy balance. By sensing BA and pharmacological agonists such as INT-777 or oleanolic acid, TGR5 on L-cells drives the secretion of GLP-1 and PYY, hormones that promote insulin release, suppress appetite, and boost EE. Gut microbiota-mediated increases in taurine-conjugated BA species after bariatric procedures further amplify TGR5 signaling, contributing to the improvements in glucose homeostasis, lipid metabolism, and adaptive thermogenesis. Thus, targeting gut TGR5, either directly with selective agonists or indirectly via microbiome modulation, holds considerable promise as a therapeutic strategy for obesity and related metabolic disorders.

### 4.6. Endocrine Pancreas

Pancreatic islets are micro-organs embedded within the exocrine pancreas, comprising diverse endocrine cell types that secrete hormones essential for maintaining glucose homeostasis. Rodent islets generally comprise about 75% to 80% beta cells and 15% to 20% alpha cells, while human islets present fewer beta cells (55% to 75%) and more alpha cells (30–45%) [93]. Insulin secretion by beta cells plays a critical role in energy homeostasis, primarily by promoting glucose uptake [94] and satiety [95].

Activation of TGR5 can regulate pancreatic beta and alpha cells to control hormone secretion [22,96,97], as its expression has been observed in both alpha and beta cells [96,98]. The activation of TGR5 with the selective ligand oleanolic acid augmented cytosolic Ca2+ oscillations and both basal and stimulated insulin secretion in a dose-dependent manner. Also, when LCA was tested, it caused an increase in cytosolic Ca2+ oscillations and glucose-mediated insulin secretion in both MIN6 cells and human islets, while INT-777 augmented insulin release in human islets under both basal and stimulated conditions. The mechanism underlying TGR5 activation by oleanolic acid and INT-777 involves the stimulation of adenylyl cyclase activity and induces cAMP increase above basal levels [96]. Thus, similar to TGR5 activation in ileal cells, the insulin release in response to oleanolic acid in pancreatic beta cells appears to be mediated by activation of PKA and EPAC pathways [96].

One of the TGR5 ligands, TUDCA, was capable of stimulating insulin secretion in a glucose-dependent manner in isolated mice pancreatic islets [99]. Following the TGR5 signaling pathway, the TUDCA exposure enhanced PKA and cAMP response element-binding protein (CREB) phosphorylation in a time-dependent manner, which indicates TGR5 activation via cAMP/PKA pathway to directly induce insulin secretion by beta cells [99]. In the same report, the use of INT-777 was capable of potentiating glucose-stimulated insulin secretion. Notably, the effects of TUDCA were abolished when treated with a cAMP competitor or a PKA inhibitor [99].

Contributing to the direct activation of TGR5 in insulin secretion, a study showed that the receptor activation also participates in increasing GLP-1 release from alpha cells to modulate beta cell mass and function through a paracrine mechanism [22]. They showed that treatment of αTC1-6 cells, db/db mouse islets, and human islets with the TGR5 agonist INT-777 promoted a switch from glucagon to GLP-1 production in mouse and human pancreatic alpha cells [22]. Both glucagon and GLP-1 originate from the same precursor, proglucagon, through the action of prohormone convertase-2 (PC2) and prohormone convertase-1 (PC1), respectively. Activation of TGR5 by INT-777 boosts intracellular cAMP levels, leading to PKA and CREB activation, which in turn upregulates PC1 expression. This shift promotes the cleavage of proglucagon toward GLP-1 production [22]. Besides that, TGR5 activation by the agonist INT-777, administered to hyperglycemic db/db mice, increased total islet size, beta cell area, and proliferation within the islets [22]. Therefore, TGR5 activation facilitates interaction between alpha and beta cells by promoting a shift from glucagon to GLP-1 production, thereby restoring beta cell mass and improving glucose metabolism. These findings highlight TGR5 as a promising therapeutic target for diabetes management.

Indirectly, TGR5 activation of GLP-1 release from ileal cells seems also to induce beneficial effects in pancreatic beta cells by enhancing insulin secretion and improving glucose intolerance in mice models [31,33,90,100] and in type 2 diabetic humans [101]. The rectal administration of TCA in obese type-2 diabetic (T2D) individuals was associated with increased plasma concentrations of active GLP-1. Concomitantly, an increase in plasma insulin concentrations was also observed, with a consistent plasma glucose concentration decrease [101]. A similar GLP-1 effect in insulin secretion in humans was previously observed, where a synthetic human GLP-1 stimulated insulin secretion in T2D patients and normal subjects, while a diminished pancreatic glucagon release was observed [102], being the one of the first studies to point out whether GLP-1 receptor agonists could be used pharmacologically to reduce blood glucose levels in type-2 diabetic patients [102].

## 5. Clinical Approaches

Despite several promising preclinical studies demonstrating the beneficial effects of TGR5 activation, its potential in clinical settings remains poorly explored. One of the major barriers is the pharmacokinetic profile of TGR5 agonists, including poor bioavailability, rapid metabolism, or systemic exposure that leads to undesirable off-target effects, such as gallbladder filling [11,35,103]. Additionally, the tissue specificity of TGR5 activation is crucial and its systemic activation may trigger unwanted effects in non-target tissues, whereas selective activation in metabolic or intestinal tissues remains challenging. Moreover, species-specific differences in BA composition and TGR5 distribution limit the direct translation of findings from rodent models to humans. These factors collectively highlight the need for more refined, tissue-targeted agonists and improved translational models that better reflect human physiology.

To date, only two studies registered on ClinicalTrials.gov (NCT) have specifically focused on TGR5 activation, both using SB-756050 in healthy and type 2 diabetic individuals. Other clinical trials have also investigated BAs, such as CDCA and UDCA, which act as potent TGR5 activators in the context of metabolic dysregulation. Ongoing registered clinical trials evaluating TGR5 activators as therapeutic agents for metabolic disorders are summarized in Table 2.

Hodge and colleagues were the first to describe the administration of a selective TGR5 agonist, SB-756050 (GlaxoSmithKline), in patients with T2D [35]. In this study, SB-756050 was safe, readily absorbed, and well tolerated. However, it exhibited nonlinear pharmacokinetics and patient-dependent pharmacodynamic effects [35]. Additionally, a clinical study demonstrated that low-dose UDCA intake enhances GLP-1 secretion through TGR5 signaling in patients with T2D [104], representing the first evidence in humans of increased GLP-1 levels and reduced blood glucose in response to UDCA.

Many clinical approaches aimed at combating obesity have focused on medications that regulate appetite and EE [105]. However, several of these anti-obesogenic drugs have been linked to serious adverse effects, including pulmonary vascular disease and heart complications [52,106]. As a result, there is a pressing need for safer appetite suppressants with minimal side effects. Given the prominent role of TGR5 activation in promoting hypophagia and body weight loss in preclinical models, and its association with increased EE in healthy individuals [107], TGR5 is increasingly recognized as a compelling target for therapeutic intervention. Notably, early studies using exogenous TGR5 agonists indicate that its activation is well tolerated in humans, further reinforcing its potential for safe and effective obesity treatment.

The results of ongoing clinical studies will be critical for assessing the safety, efficacy, and bioavailability of both endogenous and exogenous TGR5 activators in humans. If the promising effects observed in mice, such as body weight reduction and increased EE, can be replicated in humans, it could open new therapeutic avenues for treating obesity and T2D.

## 6. Limitations, Challenges, and Controversies

A key concern regarding systemic TGR5 activation is its ubiquitous expression, which makes achieving tissue-specific effects challenging. One way to address this issue is through targeted delivery systems, such as nanoparticles or liposomes, which can help direct the agonist to specific organs or tissues [108]. These tissue-specific approaches may mitigate most off-target effects associated with whole-body TGR5 activation.

Risks associated with systemic TGR5 activation include cardiovascular effects and disturbances in biliary function. A previous study showed that excessive TGR5 activation can promote cardiac hypertrophy in mice [109], raising concerns about the safety of broad systemic stimulation. Conversely, other research demonstrated cardioprotective effects of TGR5 activation, including anti-inflammatory and metabolic benefits that may support cardiovascular health [110]. Notably, the study reporting cardiac damage in mice employed a chemical strategy to increase systemic BA levels, thereby activating not only TGR5 but also other BA receptors through their respective agonists. In contrast, the study showing cardioprotective effects used a more physiologically relevant intervention: dietary supplementation with the specific TGR5 activator INT-777 [109], which typically results in lower systemic exposure due to gradual absorption. Given that cardiomyocytes express multiple BA receptors, such as FXR and sphingosine-1-phosphate receptor 2 (S1PR2) [111], the observed cardiac damage could be mediated through the activation of a different BA receptor. These contrasting findings emphasize the importance of dosing, receptor specificity, and cellular context when evaluating the safety and therapeutic potential of TGR5-targeted therapies.

Supporting this notion, high doses of TGR5 agonists have also been linked to biliary side effects. For example, administration of LCA or INT-777 at 60 mg/kg has been shown to induce excessive gallbladder filling and distension in mice, raising concerns about potential disruptions in biliary physiology [112]. These observations further highlight that while TGR5 activation holds therapeutic promise, systemic and high-dose stimulation may impair normal organ function. Therefore, future drug development must prioritize tissue selectivity, receptor specificity, and careful dose optimization to minimize off-target effects and organ-specific toxicity.

Another important factor influencing TGR5-mediated effects is the alteration of gut microbiota induced by metabolic disorders, such as T2D, nonalcoholic fatty liver disease, and obesity. These conditions impact both microbiome composition and BA profile [113,114], which is particularly relevant given that several BAs depend on microbial activity for their formation, such as the secondary bile acids LCA and DCA, which are converted from primary BAs through bacterial 7α-dehydroxylation [115]. Additionally, gut bacteria mediate the epimerization of CDCA into UDCA, which in turn can be conjugated with taurine to form TUDCA [116].

Beyond these interactions, the gut microbiome varies significantly across mammalian species [117], influenced by factors such as host phylogeny, diet, and gut morphology [117,118]. As a result, findings from murine models regarding TGR5 activation cannot be directly translated to humans due to differences in BA profiles [119]. Furthermore, a comparative study has demonstrated variations in TGR5 expression levels across different tissues in humans, mice, and other mammals [120], further emphasizing the need for species-specific considerations in TGR5-related research.

It is important to note that the majority of the studies discussed here were conducted in male animals, and the results may differ in females due to the influence of female sex hormones and physiological fluctuations associated with the estrous cycle on metabolism [121,122]. For instance, female TGR5 knockout mice on HFD demonstrated accelerated body weight gain and increased body fat than wild-type mice, whereas TGR5 knockout in males did not affect these parameters [15]. Additionally, female mice had higher total serum BA concentrations when fed six of nine different diets compared to male mice [123]. However, unconjugated bile acids were higher in male mice under specific dietary conditions [123]. Female mice also exhibited increased total and primary serum BA levels, regardless of microbiota presence [16]. Moreover, gender-dependent effects of TGR5 gene deletion were observed in the metabolic profiles of DIO mice [17]. Collectively, these findings underscore the need for further research to elucidate the mechanisms underlying the sex-dependent effects of TGR5 activation.

## 7. Conclusions and Future Directions

**Figure 2:**
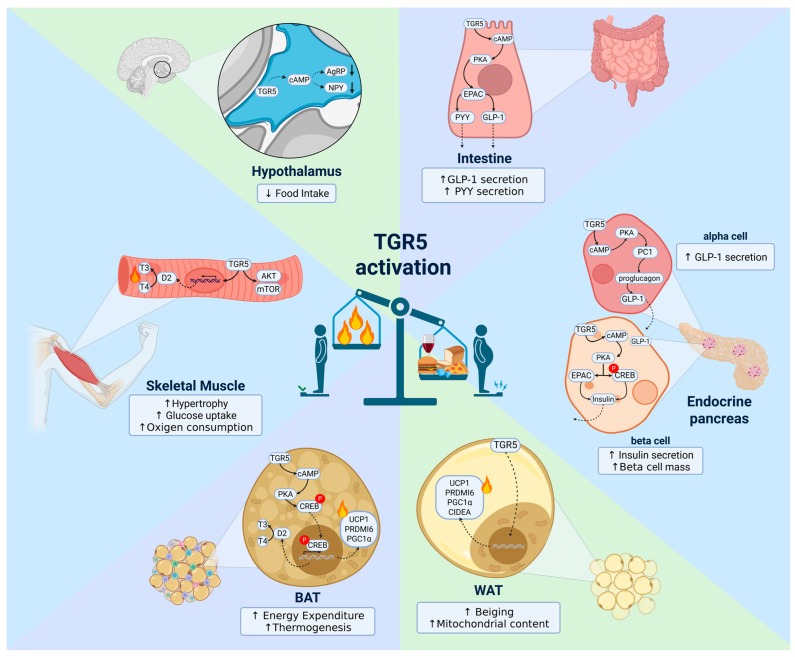
Pleiotropic effects mediated by TGR5 activation on energy homeostasis regulation. TGR5 activation triggers distinct signaling pathways in various tissues, regulating energy balance and glucose metabolism. In the hypothalamus, activation of TGR5 increases intracellular cAMP levels, which leads to the suppression of the orexigenic neuropeptides AgRP and NPY. This downregulation contributes to reduced food intake. In the intestine, activation of TGR5 stimulates the cAMP/PKA/EPAC signaling pathway in enteroendocrine L-cells, leading to enhanced secretion of the incretin hormone GLP-1 and the satiety hormone PYY. These gut-derived hormones contribute to improved glucose homeostasis and reduced appetite. In the endocrine pancreas, TGR5 activation engages the cAMP/PKA/EPAC/CREB signaling axis, leading to enhanced insulin secretion from beta cells and increased GLP-1 secretion from alpha cells. GLP-1 secreted by alpha cells can also act in a paracrine manner to further stimulate insulin secretion from neighboring beta cells. In skeletal muscle, TGR5 activation promotes the AKT/mTORC1 signaling pathway and induces the expression of D2, which catalyzes the local conversion of T4 to the more active T3. This increase in intracellular T3 levels enhances glucose uptake, oxygen consumption, and protein synthesis, ultimately contributing to improved metabolic function and muscle hypertrophy. In BAT, TGR5 activation stimulates the cAMP/PKA/CREB signaling pathway, leading to increased expression of UCP1. This upregulation enhances mitochondrial uncoupling and thermogenic activity, resulting in elevated heat production and increased energy expenditure, thereby contributing to the regulation of body weight and metabolic homeostasis. In WAT, TGR5 activation promotes beiging and mitochondrial biogenesis through upregulation of UCP1, PRDM16, PGC1α, and CIDEA. These molecular changes enhance oxidative metabolism and thermogenic capacity, contributing to increased energy expenditure and improved metabolic health. Collectively, TGR5 activation orchestrates a multifaceted regulatory network across tissues, serving as a critical mediator of metabolic homeostasis and energy balance through its effects on hormonal, metabolic, and thermogenic pathways. AgRP: Agouti-related peptide; AKT: Protein kinase B; BAT: Brown adipose tissue; cAMP: Cyclic adenosine monophosphate; CIDEA: Cell death-inducing DNA fragmentation factor alpha-like effector A; CREB: cAMP response element-binding protein; D2: Type 2 iodothyronine deiodinase; EPAC: Exchange protein directly activated by cAMP; GLP-1: Glucagon-like peptide 1; mTOR: Mammalian target of rapamycin; NPY: Neuropeptide Y; T3: Triiodothyronine; T4: Thyroxine; PGC1α: Peroxisome proliferator-activated receptor gamma coactivator 1-alpha; PKA: Protein kinase A; PRDM16: PR domain containing 16; PYY: Peptide YY; T3: Triiodothyronine; TGR5: Takeda G protein-coupled receptor 5; UCP1: Uncoupling protein 1; WAT: White adipose tissue. Arrows ↑ and ↓ indicate increase and decrease, respectively. Created in Biorender. Helena Barbosa. (2025) https://BioRender.com (accessed on 8 May 2025).

Extensive research has highlighted the beneficial effects of TGR5 activation on body weight reduction in experimental obesity models, primarily by enhancing EE and thermogenesis as well as reducing food intake. Additionally, TGR5 activation improves glucose metabolism, mitigates macrophage-driven inflammation in adipose tissue, and reduces hepatic lipid accumulation, highlighting its significant potential as a therapeutic target for several aspects of metabolic dysregulation. The main effects of TGR5 activation on energy homeostasis are illustrated in Figure 2.

Although targeting BA signaling pathways via TGR5 offers significant promise for metabolic regulation, several challenges remain. Identifying tissue-specific TGR5 targets will be essential for enhancing efficacy, improving specificity, and minimizing potential side effects. Moreover, most preclinical studies have utilized non-physiological concentrations of TGR5 activators, highlighting the need for further research under conditions that better reflect physiological relevance. Future studies should focus on developing selective TGR5 modulators, evaluating long-term safety profiles, and translating these findings into clinical applications to determine whether TGR5-based therapies can effectively combat metabolic disorders in humans.

## Figures and Tables

**Table 1:** EC₅₀ values of the main TGR5 agonists.

TGR5 Agonist	EC50 (μM)	Reference
LCA	0.53	[32]
DCA	1.25	[30]
CDCA	6.71	[30]
CA	13.6	[30]
UDCA	36.4	[30]
TLCA	0.33	[32]
TDCA	0.79	[30]
TCDCA	1.92	[30]
TCA	4.95	[30]
TUDCA	30.0	[30]
INT-777	0.82	[33]
BAR501	1.0	[34]
SB-756050	1.3	[35]
Oleanolic acid	2.25	[36]
Obacunone/Liminoid	Not specified	

LCA: Lithocholic acid, DCA: Deoxycholic acid, CDCA: Chenodeoxycholic acid, CA: Cholic acid, UDCA: Ursodeoxycholic acid, TLCA: Taurolithocholic acid, TDCA: Taurodeoxycholic acid, TCDCA: Taurochenodeoxycholic acid, TCA: Taurocholic acid, TUDCA: Tauroursodeoxycholic acid, INT-777: Synthetic TGR5 agonist, BAR501: Synthetic TGR5 agonist, SB-756050: Synthetic TGR5 agonist.

**Table 2:** Ongoing registered clinical trials evaluating TGR5 activators as therapeutic agents for metabolic disorders.

TGR5 Agonist	Condition	Study Title	Clinical Trials Identifier:	Study Design, Interventions, and Location
SB756050 (GlaxoSmithKline)	Type 2 Diabetes Mellitus	A Study to Test How SB756050 Affects Subjects With Type 2 Diabetes Mellitus After 6 Days of Dosing	NCT00733577	Study Type: Interventional Enrollment: 48 participants Phase: 1 Doses: 15 to 600 mg Duration: 6 days Placebo-Controlled Location: USA
SB756050 (GlaxoSmithKline)	Type 2 Diabetes Mellitus	First-Time-in-Humans Study to Assess Safety, Pharmacokinetics & Pharmacodynamics of SB756050	NCT00607906	Study Type: Interventional Enrollment: 36 participants Phase: 1 Doses: 5–100 mg Duration: single dose Placebo-Controlled Location: USA
Bile acid CDCA and oleanolic acid	Healthy Volunteers	Effect of Bile Acids on the Secretion of Satiation Peptides in Humans	NCT01674946	Study Type: Interventional Enrollment: 12 participants Phase: 1 Doses: Oleanolic acid: 1–20 mM/L; CDCA: 5–15 mM/L Duration: single intraduodenal perfusion Placebo-Controlled Location: Switzerland
Bile acid CDCA	Type 2 Diabetes Mellitus	Effect of Bile Acids on GLP-1 Secretion	NCT01666223	Study Type: Interventional Enrollment: 20 participants Phase: Not Applicable Doses: 1250 mg Duration: single intraduodenal perfusion Placebo-Controlled Location: Denmark
Bile acid CDCA	Metabolic Syndrome	Effects of FXR Activation on Hepatic Lipid and Glucose Metabolism	NCT00465751	Study Type: Interventional Enrollment: 30 participants Phase: 1 Doses: 500 mg Duration: 3 months Placebo-Controlled Location: Switzerland
Bile acid UDCA	Type 2 Diabetes Mellitus	Efficacy of Ursodeoxycholic Acid (UDCA) in Patients With Type 2 Diabetes	NCT05416580	Study Type: Interventional Enrollment: 60 participants Phase: 3 Doses: 1500 mg Duration: 8 weeks Placebo-Controlled Location: Bosnia and Herzegovina
Bile acid UDCA (Ursodiol)	Obesity and Type 2 Diabetes Mellitus	Ursodiol on Insulin Sensitivity, Gastric Emptying, and Body Weight With Type 2 Diabetes on Metformin	NCT02033876	Study Type: Interventional Enrollment: 24 participants Phase: 2 Doses: 600 mg twice daily Duration: 2 weeks Placebo-Controlled Location: USA
Bile acid TUDCA	Obesity	Effect of Endoplasmic Reticulum Stress on Metabolic Function (TUDCA/PBA)	NCT00771901	Study Type: Interventional Enrollment: 101 participants Phase: Not Applicable Doses: 1750 mg/day Duration: 4 weeks Placebo-Controlled Location: USA
Bile acid TUDCA	Type 2 Diabetes Mellitus	Effects of Dietary Supplement Tauroursodeoxycholic Acid on Vascular Function	NCT03331432	Study Type: Interventional Enrollment: 8 participants Phase: Not Applicable Doses: 1750 mg/day Duration: 4 weeks Location: USA
Oleanolic acid (olive oil)	Metabolic Diseases	Prevention With Oleanolic Acid of Insulin Resistance (PREOLIA)	NCT05049304	Study Type: Interventional Enrollment: 22 participants Phase: Not Applicable Doses: Functional olive oil enriched in Oleanolic acid Duration: single intake Location: Spain
Oleanolic acid (olive oil)	Type 2 Diabetes Mellitus	Oleanolic Acid as Therapeutic Adjuvant for Type 2 Diabetes Mellitus (OLTRAD STUDY)	NCT06030544	Study Type: Interventional Enrollment: 100 participants Phase: 2 Doses: 55 mL/day of a functional olive oil enriched in Oleanolic acid Duration: 1 year Location: Spain

CDCA: chenodeoxycholic acid; UDCA: ursodeoxycholic acid; TUDCA: tauroursodeoxycholic acid.
